# Conservation and Divergence of the Trihelix Genes in Brassica and Expression Profiles of *BnaTH* Genes in *Brassica napus* under Abiotic Stresses

**DOI:** 10.3390/ijms232415766

**Published:** 2022-12-12

**Authors:** Cuiping Zhang, Lijing Lu, Ruolin Gong, Xing Su, Fengbo Liu, Ru Zhang, Jihong Hu

**Affiliations:** State Key Laboratory of Crop Stress Biology for Arid Areas, College of Agronomy, Northwest A&F University, Xianyang 712100, China

**Keywords:** trihelix, transcription factor, brassica, evolution, abiotic stress, drought

## Abstract

Trihelix (TH) proteins are a family of plant-specific transcription factors that play a role in light response and are extensively involved in plant growth and development, as well as in various stress responses. However, the function of *TH* genes in *Brassica napus* (*B. napus*) remains unclear, as does the evolution and differentiation pattern of *TH* genes in Brassica plants. Here, we identified a total of 455 *TH* genes in seven species, including six Brassica species and *Arabidopsis*, which were grouped into five clades, GT-1, GT-2, GTγ, SH4, and SIP1, each with 69, 142, 44, 55, and 145 members, respectively. The types and distributions of motifs of the TH proteins and the structures of the *TH* genes are conserved in the same subgroup, and some variations in certain amino acid residues occur in *B. napus* when inheriting motifs from *Brassica rapa* (*B. rapa*) and *Brassica oleracea* (*B. oleracea*). Collinearity analysis revealed that the massive expansion of *TH* genes in tetraploid species was attributed to the hetero-tetraploidization of diploid ancestors and gene duplication events within the tetraploid species. Comparative analysis of the membership numbers of five subgroups in different species revealed that the GT-2 and SIP1 genes underwent significant expansion during evolution, possibly to support the better adaptation of plants to their environments. The differential expression of the *BnaTH* genes under five stresses indicates that the *BnaTH* genes are involved in plant responses to stresses such as drought, cold, and heat. The presence of different stress-responsive *cis*-elements in the upstream promoter region of the genes indicated that *BnaTH* genes have the potential to cope with variable environments. Meanwhile, qRT-PCR analyses also confirmed that five *TH* genes respond to different abiotic stresses. Our results provide information and candidates for further studies on the role of *TH* genes in stress resistance of *B. napus*.

## 1. Introduction

Trihelix (TH) transcription factors, as a key regulatory factor, were originally called GT elements due to their specific binding to GT elements, which is required for light-responsive transcription [1,2]. TH factors contain a conserved trihelix DNA-binding domain characterized by a typical trihelix (helix–loop–helix–loop–helix) structure, which binds specifically to GT elements in the promoters of target genes [3,4] and plays a pivotal role in a variety of developmental processes and environmental stress responses. Based on its conserved domains and evolutionary relationships, the TH gene family is divided into five clades, namely GT-1, GT-2, SH4, SIP1, and GTγ. Studies have revealed that GT factors, which have a trihelix structure that resembles the Myb/SANT-LIKE DNA-binding domain, likely originated from an MYB-like gene carrying only one repeat [3]. However, the recognition sequences of the GT transcription factor and the MYB protein are different, which may be caused by the insertion of gaps between the two helices [3].

In recent years, with the completion of the genome assembly of various species, the genome-wide identification of the TH gene family has been reported in many species. In total, 30 and 31 *TH* genes were identified in *Arabidopsis* and rice, respectively [2,5]. In addition, 31, 35, 94, 52, 56, 52, 20, and 36 *TH* genes were identified in *Fagopyrum esculentum*, *Moso bamboo*, *Triticum aestivum*, *Gossypium arboreum*, *Populus trichocarpa*, *B. rapa*, *Chrysanthemum*, and *Solanum lycopersicum*, respectively [6,7,8,9,10,11,12,13]. Recent studies have revealed that TH factors not only respond to light stress and regulate the expression of light-responsive genes but are also involved in abiotic stress responses [2,14]. For instance, the TH family gene *ShCIGT* mediates cold tolerance and drought tolerance by interacting with SnRK1 in tomato [15]. Overexpression or knockout of the *AST1* in *Arabidopsis* could positively influence tolerance to salt, osmotic, and drought stress [16]. *BnSIP1-1* of *B. napus* has been proved to be capable of coping with various abiotic stresses, and the overexpression of *BnSIP1-1* can improve the germination rate of *B. napus* under osmotic and salt stresses [17]. *Arabidopsis*, AtGT2L, as a Ca^2+^-dependent CaM-binding protein, is strongly induced to express under cold and salt stresses [18]. However, the function of TH family genes in Brassica species and their roles in abiotic stresses have not been systematically reported. Thus, it is essential to systematically characterize the TH family genes in Brassica species, to explore their evolutionary relationships among species, and to further investigate their roles under abiotic stresses.

*B. napus* (AnAnCnCn, 2n = 38), a member of the Brassica species, is an allopolyploid that was formed by hybridization between *B. rapa* (AnAn, n = 10) and *B. oleracea* (CnCn, n = 9) about 7500 years ago [19,20]. In addition, the Brassica genus also includes *Brassica nigra* (*B. nigra*; BnBn, n = 8), along with two allotetraploids, *Brassica juncea* (*B. juncea*; AnAnBnBn, 2n = 36) and *Brassica carinata* (*B. carinata*; BnBnCnCn, 2n = 34) [21,22]. The evolutionary relationships of these six Brassica species are known as the classic U’s triangle model [23]. Studies have confirmed that the Brassica species *B. nigra*, *B. rapa*, *B. oleracea*, *B. juncea*, *B. carinata*, and *B. napus* have undergone a whole-genome triplication (WGT) since their divergence from the *Arabidopsis* species [24,25]. The genome underwent chromosomal rearrangement, gene loss, and the divergence of retained paralogues after WGT, with substantial gene loss occurring in an asymmetric and reciprocal manner [26]. A large number of over-retained genes after WGT belongs to the gene families that underlie adaptation to environmental factors such as salt, cold, osmotic stress, light, wounding, pathogen defense, and so on [24]. To date, whole genome sequencing of six species in the U’s model has been completed. Plant growth and development are accompanied by adaptation to the environment, which is always complex and changeable. Therefore, exploring genes such as *TH* genes, and identifying their roles in abiotic stresses, will provide insights into improving crop yields and environmental adaptation.

*B. napus* is an important oilseed crop, and the production of rapeseed is second only to that of soybeans in terms of the worldwide production of oilseed crops. However, with continuous changes in the global climate, environmental factors such as drought and low temperatures have become the main limiting factors for rapeseed production, resulting in a decline in rapeseed yields and quality. Although many breeding works have used heterosis to screen and breed varieties with high yields and oil content, the research into abiotic stress in rapeseed is still relatively underdeveloped. Therefore, it is urgent to cultivate stress-resistant *B. napus* varieties capable of adapting to the changing environment in order to ensure yield and quality.

In our study, we identified and characterized the TH transcription factors in six Brassica species and expression patterns in *B. napus* under different abiotic stresses. The evolution of the TH gene family was investigated in Brassica genus. To establish the role of the *TH* genes in rapeseed, we evaluated their expression patterns in different tissues and under different abiotic stresses, including drought, cold, and heat. Our research provides new insights into enhancing stress resistance in *B. napus* with the goal of improving yield and quality.

## 2. Results

### 2.1. Identification of TH Genes in Seven Species

A total of 455 *TH* genes was identified in seven species, with 29, 51, 49, 54, 92, 86, and 94 in *Arabidopsis*, *B. nigra*, *B. rapa*, *B. oleracea*, *B. juncea*, *B. carinate*, and *B. napus*, respectively. All *TH* genes were subsequently renamed according to their species and chromosomal locations (Table S1), yielding *AtTH-1* to *-29*, *BniTH-1* to *-51*, *BraTH-1* to *-49*, *BolTH-1* to *-54*, *BjuTH-1* to *-92*, *BolTH-1* to *-86*, and *BraTH-1* to *-94*. Next, the physicochemical properties of the TH proteins, including protein length, molecular weight (MW), theoretical isoelectric point (pI), instability index (II), aliphatic index (AI), and the grand average of hydropathicity (GRAVY), were calculated to provide further information (Table S2). The length of TH proteins varied from 109 (BolTH-35) to 1149 (BnaTH-19) amino acids (aa), with an MW range of 12,940.84 (BolTH-35) –27,364.53 (BolTH-18) kDa. The pI values were between 4.65 (AtTH-18) and 10.83 (BniTH-44), and the II ranged from 28.75 (BraTH-4) to 86.3 (BcaTH-32). The AI can vary from 38.53 (BolTH-35) to 87 (BcaTH-51). All the TH proteins presented GRAVY values of <0, ranging from −0.357 (BcaTH-42) to −1.358 (BcaTH-51), implying their hydrophilic characterization. Subcellular localization revealed that nearly all the TH proteins (389, 85.5%) were located in the nucleus. This result is consistent with their roles as transcription factors. In addition, small proportions of the proteins are located in other regions, such as the chloroplast (22, 4.8%), cytoplasm (34, 7.5%), peroxisome (8, 1.8%), endoplasmic reticulum (1, 0.2%), and vacuole (1, 0.2%).

### 2.2. Phylogenetic Analysis and Classification of TH Genes

**Figure 1:**
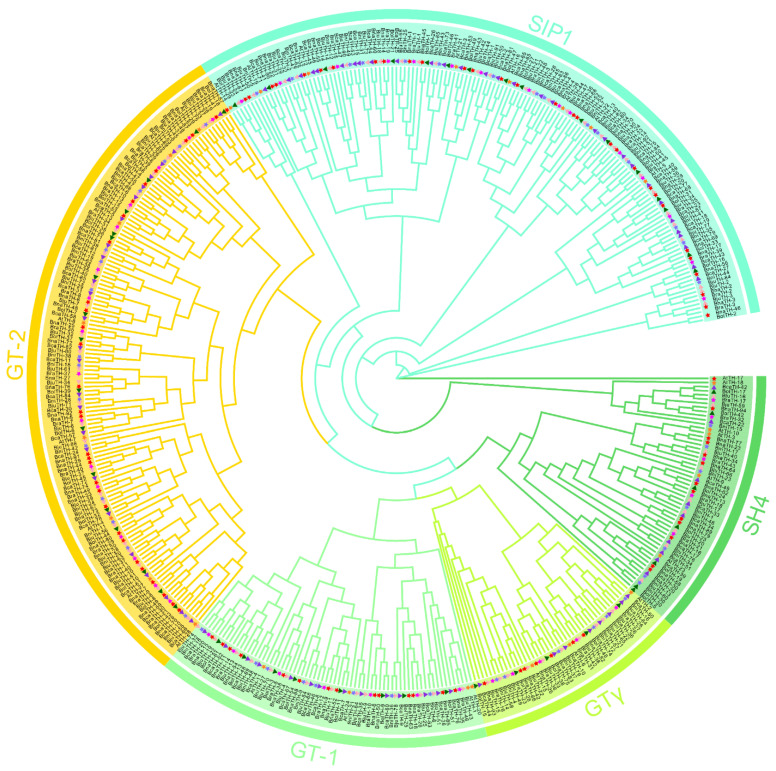
Phylogenetic tree using 455 TH proteins from 7 species, including *Arabidopsis*, *B. carinate*, *B. juncea*, *B. napus*, *B. oleracea*, *B. nigra*, *B. oleracea*, and *B. rapa*, marked as yellow pentagram, purple triangle, pink triangle, red pentagram, purple pentagram, green triangle, and purple pentagram, respectively. The clades of group GT-1, GT-2, GTγ, SH4, and SIP1 are distinguished by different colors.

To uncover the phylogenetic relationships of the *TH* genes, we constructed an unrooted phylogenetic tree based on the multiple sequence alignment of 455 TH proteins among the seven species, namely *Arabidopsis*, *B. nigra*, *B. juncea*, *B. carinate*, *B. rapa*, *B. napus*, and *B. oleracea* (Figure 1). These *TH* genes were classified into five clades, namely GT- 1, GT-2, GTγ, SH4, and SIP1 [11], containing 69, 142, 44, 55, and 145 *TH* genes, respectively. The SIP1 clades contained the most *TH* members (145, 31.9%), while the GTγ clades had the fewest *TH* genes (44, 9.7%). These results indicated that the number of *TH* genes is uneven across the five subgroups.

### 2.3. Gene Structure and Protein Conservative Domain Analysis

Through MEME domain analysis, we identified 10 distinct motifs for the structural diversification of 455 *TH* genes, which we refer to as motifs 1 to 10 (Figure S1 and Table S3). Overall, similar motif compositions and distributions were found in the same subgroups, whereas they differed significantly among the subgroups. All subgroup members contain about three motifs, except the GT-2 subgroup, which has about 10 motifs. All TH proteins contain motif 1, whereas motif 3 is almost unique to GT-2. Furthermore, the GT-2 clade with the most abundant motif types, contains all of the motifs except for motif 4 and motif 9, and most members contain two of motif 1, two of motif 2, and two of motif 3. In addition, motif 4 which has the longest sequence, is mostly present in GT-1 and only appears a few times in SIP1. These results suggested that subgroup-specific motifs might play pivotal roles in specific functions. Gene structure analysis showed that the exon lengths of genes in the same subgroup were diverse, but the number was almost always conserved (Figure S1). For example, most GTγ and SIP1 members contain one exon, and GT-1 and SH4 members contain 2–5 exons. In contrast, the GT-2 subfamily has a more complex exon number and length than the others.

**Figure 2:**
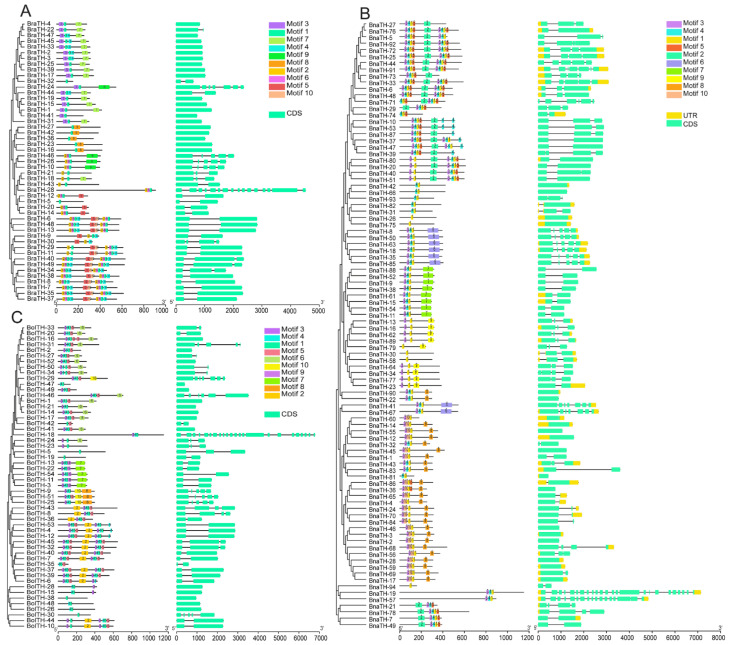
Comparative analysis of conserved domains and gene structures of *BraTH* genes, *BolTH* genes, and *BnaTH* genes. The colored boxes with numbers indicate the different conserved motifs identified by MEME. The scale bar at the bottom estimates the lengths of the motifs, exons, and introns. (**A**) Phylogenetic relationships, conserved protein motifs, and gene structures of 49 *BraTH* genes. (**B**) Phylogenetic relationships, conserved protein motifs, and gene structures of 94 *BnaTH* genes. (**C**) Phylogenetic relationships, conserved protein motifs, and gene structures of 54 *BolTH* genes.

In order to explore the gene structural changes in *B. napus* during evolution, we performed structural analysis for *B. napus* with its diploid progenitors *B. rapa* and *B. oleracea*. We separately predicted ten motifs in these three species and compared their amino acid residues (Figure 2, Table S4). We found that most motifs in *B. napus* were similar to those in *B. rapa* and *B. oleracea*. In particular, the amino acid residues of motif 1 and motif 6 were almost identical to their corresponding sequences in the progenitors, indicating that these two motifs were highly conserved during rapeseed evolution. In addition, we found that some motifs in *B. napus* were more likely to be derived from *B. rapa*. For example, motif 5 in *B. napus* is more similar to motif 4 in *B. rapa* than motif 5 in *B. oleracea*, and motif 4 in *B. napus* is more likely to have originated from motif 6 in *B. rapa*. Furthermore, the orthologous among the three species have similar gene structures and motif features (Figure 2 and Table S5). For instance, *BnaTH-57* has similar exons with its homologs *BraTH-28* and *BolTH-18*; additionally, the composition and distribution of motifs in protein sequences and the amino acid residues in the motifs are identical. Moreover, there is a unique motif 9 in *B. napus*, which may have been newly formed during *B. napus* evolution.

Moreover, we found that some BraTH motifs originated from only one species, such as BnaTH-11, which has the orthologs BraTH-14 and BolTH-13, whose motifs appear to be derived from BolTH-13 only, as the corresponding motif cannot be found in BraTH-14. However, most BnaTH motifs have more complex origins, and they may be derived from both *B. rapa* and *B. oleracea*, such as, BnaTH-51 and BnaTH-8. Our analysis of members of the SIP1 subgroup in *B. napus* and its diploid ancestors revealed that some BnaTHs inherited motifs from ancestors with motif insertions and deletions (Figure S2). For example, there are six motifs in BnaTH-28, while its homologs BolTH-20 and BraTH-25 have seven and five, respectively, and there are six motifs in BnaTH-46 compared to four in the homolog BolTH-2. These results implied that the evolution of *TH* genes in *B. napus* is intricate and diverse, and that they have inherited genetic material from ancestral species while undergoing a number of mutations in order to meet autogenous growth and development.

### 2.4. Cis-Acting Elements in the Promoter Region of TH Genes

In order to better understand the potential regulatory roles of *TH* genes, the *cis*-acting elements in the 1500 bp upstream region of *TH* gene promoters were predicted using PlantCARE software. Twenty-seven distinct *cis*-acting elements were identified and grouped into three main categories (Tables S6 and S7). The first one was engaged in light responsiveness, such as Box 4 elements, I-boxes, TCT motif, ATC motif, GATA motif, MRE, G-box, SP1, AE-box, ATCT-motif, MRE, TCCC-motif, chs-CMA1a, and GT1-motif; the second was associated with stress responses, including drought stress (MBS), anaerobic induction (ARE), and low-temperature stress (LTR); the third was involved in plant growth and development, including circadian (circadian control), meristem expression (CAT-box), and phytohormone responses, such as abscisic acid (ABRE), methyl jasmonate (CGTCA-motif and TGACG-motif), auxin (TGA element), salicylic acid (TCA element), and so on (Tables S6 and S7). A total of 99.3% (442/445) of genes contained light-responsive elements, with *BcaTH-15* containing the most light-responsive elements (24) and *BnaTH-66* having the least (1). Among the 168 genes featuring drought-responsive elements, *BcaTH-74* contains the most numbers of MBS (4). In addition, the most abundant of these 168 genes are members of the SIP1 subgroup (53, 31.5%), followed by the GT-1 subgroup (39, 23.2%); meanwhile, the SH4 subgroup (17, 10.1%) had the fewest members (Table S7). There are 220 genes involved in low temperature response elements; of these, members of the SIP1 subgroup account for the largest proportion (75, 34.1%), followed by GT-2 (55, 25.0%). *BolTH-43* contains 9 low-temperature-response elements, which is the most among the 220 genes. In addition, there are a large number of other elements in the *TH* genes, such as methyl-jasmonate (MeJA)-responsive elements, abscisic-acid (ABA)-responsive elements, salicylic-acid (SA)-responsive elements, auxin-responsive elements, gibberellin-responsive elements, and ethylene (Eth)-responsive elements, indicating that they may be involved in phytohormone signaling pathways (Table S7). These results suggested that *TH* genes play an important role in plant growth and response to stress resistance. Moreover, compared with other subgroups, GT-2 and SIP1 have more of these elements, implying that the gene expansions of the subgroups GT-2 and SIP1 may be associated with environmental adaptation.

### 2.5. Chromosomal Distribution and Gene Duplication Events of the TH Family

**Figure 3:**
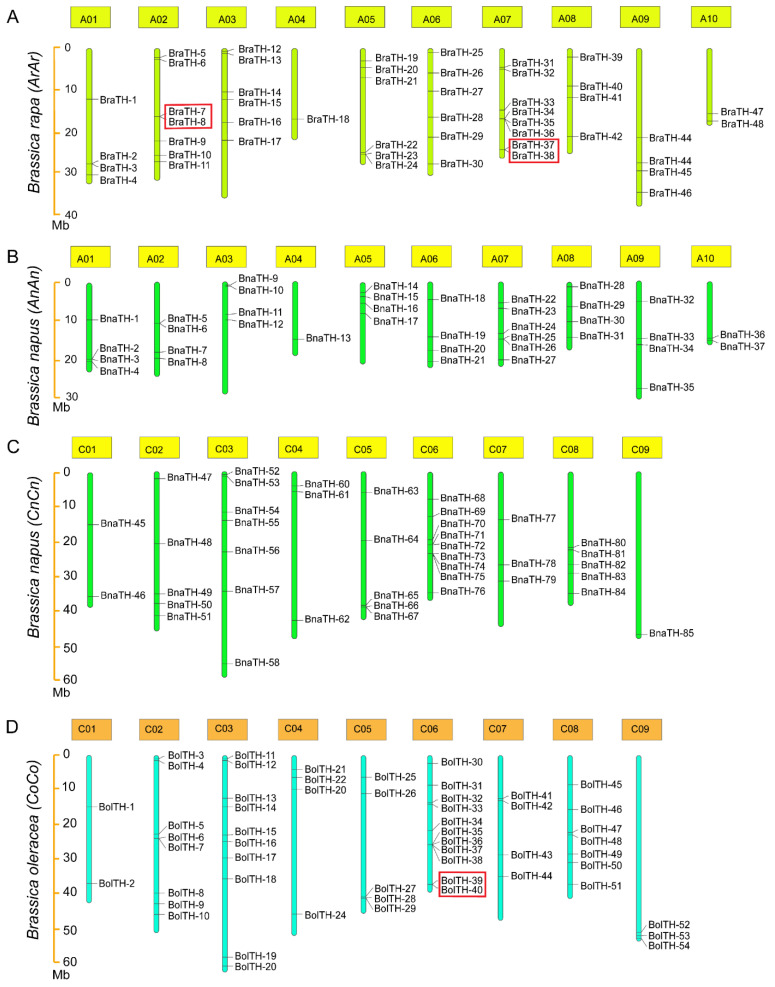
Chromosomal distribution of *BraTH* genes, *BolTH* genes, and *BnaTH* genes. The colored bars represent chromosomes, and the chromosome numbers are shown at the top of the bars. *TH* genes are labeled at the right of the chromosomes. The scale on the left side indicates the chromosome length (Mb). (**A**) Chromosomal localization of *BraTH* genes in *B. rapa*. (**B**) Chromosomal localization of *BnaTH* genes in A subgenome of *B. napus*. (**C**) Chromosomal localization of *BnaTH* genes in C subgenome of *B. napus*. (**D**) Chromosomal localization of *BolTH* genes in *B. oleracea*. Genes in the red box are tandem duplication genes.

Location information of *TH* genes from seven species was obtained according to the genome annotations (Table S1). Some genes were not accurately mapped to chromosomes because of the incomplete assembly of certain genomes. Overall, *TH* genes are unevenly distributed on the chromosomes of the respective species (Figure 3). We found that the numbers and distributions of *TH* genes located in the C subgenome of *B. napus* and *B. oleracea* were remarkably consistent. Additionally, the *TH* genes located on the A subgenome of the *B. napus* and *B. rapa* genome also have high consistency, but the similarity is relatively low. In addition, the number of *TH* genes in *B. napus* is slightly lower than that on the corresponding chromosomes of *B. rape* and *B. oleracea*. This may be due to the loss of some functionally redundant *TH* genes during the evolution of *B. napus*, or the incomplete assembly of chromosomes.

**Figure 4:**
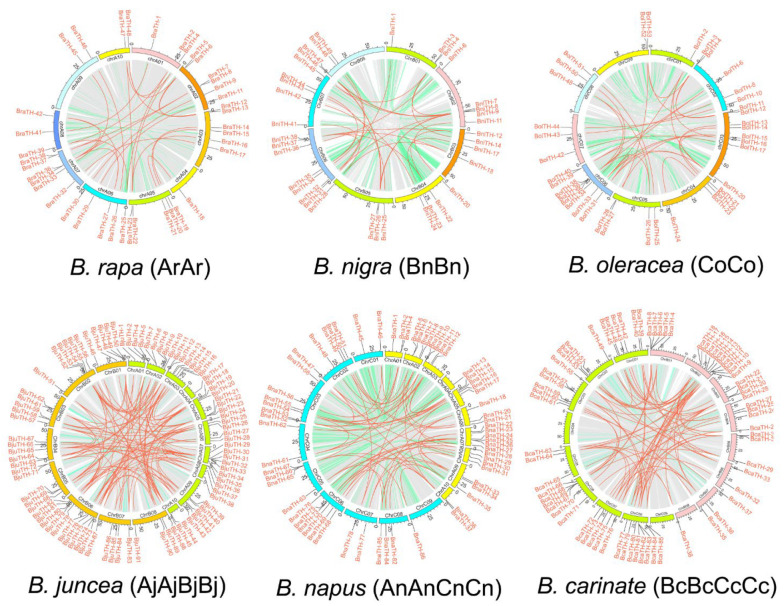
The collinearity of *TH* genes in *B. rapa*, *B. nigra*, *B. oleracea*, *B. juncea*, *B. napus*, and *B. carinate*. The green line indicates all synteny blocks between each chromosome and the red line indicates duplicated *TH* gene pairs. The chromosome number is shown at the top of each chromosome. The scale marked on the chromosome indicates the chromosome length (Mb).

The intra-species synteny relationships of *TH* genes were analyzed using the genomic information of the seven species (Figure 4, Table S8). A total of 393 paralogous pairs was identified, including 6, 90, 131, 85, 25, 29, and 27 pairs in *Arabidopsis*, *B. carinate*, *B. juncea*, *B. napus*, *B. nigra*, *B. oleracea*, and *B. rapa*, respectively. These paralogous gene pairs contained 358 *TH* genes, and most of them belong to subgroup GT-2 (110, 30.7%), followed by subgroup SIP1 (106, 29.6%). Overall, compared with the diploid species, the tetraploid species experienced more segmental duplication events. In addition, we detected nine pairs of tandem duplicated genes with 17 *TH* genes, which mainly belonged to subgroup GT-2 (Table S9).

The nonsynonymous rate (*Ka*), synonymous rate (*Ks*), and evolutionary constraint (*Ka/Ks*) ratio of duplicated gene pairs of *TH* genes were calculated (Table S8). The *Ka/Ks* ratios of all paralogous gene pairs were less than 1; 383 (98.5%) pairs were less than 0.5, and 162 (41.2%) pairs were less than 0.2, which might have encountered extremely strong purification selection after triplication, resulting in the severe inhibition of the functional differentiation of *TH* genes.

### 2.6. Evolutionary and Collinearity Analysis of TH Genes in Brassica

**Figure 5:**
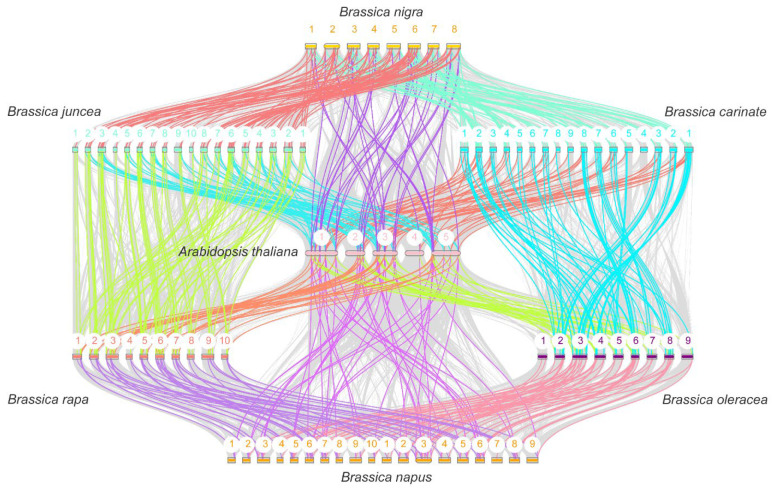
The collinearity analysis of *TH* genes between *Arabidopsis* and six Brassica species and between tetraploid and their diploid ancestors. The grey lines in the background indicate the collinear blocks in the genomes of the two species connected by the grey lines, while the colored lines highlight the syntenic *TH* gene pairs. The colored bars represent the chromosomes of the different species. The chromosome number is labeled at the top of each chromosome.

**Figure 6:**
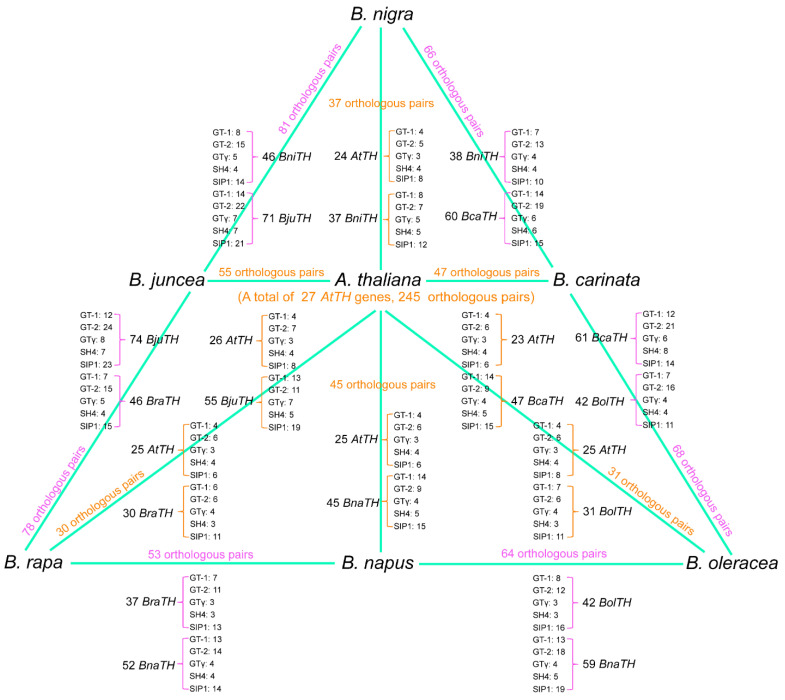
Numbers of orthologous gene pairs among different species and subfamily of orthologous genes. Orange represents the orthologous gene pair between *Arabidopsis* and Brassica species. Magenta represents the orthologous gene pair between Brassica species.

In order to trace the evolution of the TH gene family of Brassica, we analyzed the orthologous relationship between *Arabidopsis* and six Brassica species, and between the tetraploid Brassica species and their diploid ancestors (Figure 5, Table S5). A total of 655 pairs of *TH* genes showed collinearity, among which 245 pairs of orthologous genes were composed of 27 *Arabidopsis TH* genes and corresponding Brassica *TH* genes (Figure 6). The orthologous gene pairs between *Arabidopsis* and *B. rapa*, *B. oleracea*, and *B. napus* are 30, 31, and 45, respectively, indicating that some redundant genes were lost during the allo-tetraploidization of *B. napus*. In contrast, *TH* genes lost the most during the formation of the tetraploid *B. carinate*, while *B. juncea* lost the least (Figure 6). In addition, compared with the numbers of *TH* genes in tetraploid species and the numbers of orthologous genes between tetraploid species and their diploid ancestors, we observed that the *TH* genes in *B. napus* had the most variation in tetraploid species, which might differentiate into more functional genes. The number of orthologous gene pairs between *B. napus* and *B. rapa* and between *B. napus* and *B. oleracea* was 53 and 64, respectively, which may suggest that *TH* genes from *B. oleracea* are more inclined to be retained in *B. napus*.

**Figure 7:**
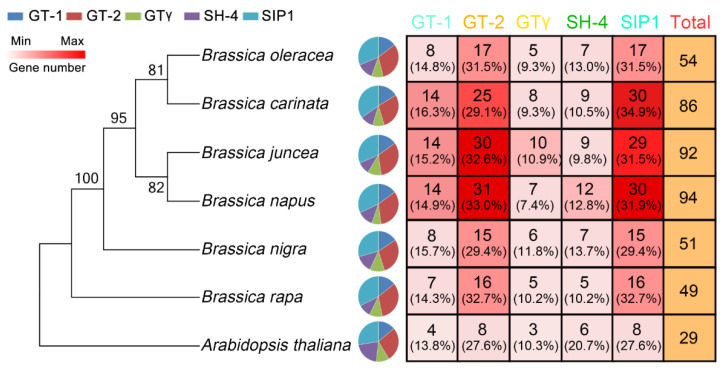
Clade distribution of *TH* genes in *Arabidopsis*, *B. rapa*, *B. nigra*, *B. oleracea*, *B. juncea*, *B. napus*, and *B. carinate*. The species tree constructed on the basis of the evolutionary relationships between the seven species is shown on the left. The subgroup distribution of *TH* genes in each species is shown in the middle pie chart. The counts and percentages of *TH* gene subgroup members in each species are given in the table on the right.

To evaluate the expansion of different clades, we investigated the relationships between *TH* genes and clades in each species (Figure 7). Overall, the GT-2 and SIP1 clades hold the largest number of *TH* genes, especially in tetraploid. However, the proportion of genes in different subgroups does not seem to change much in each species. As shown in Figure 6, the orthologous genes between *B. rapa* and *B. napus* are 37 and 52, respectively, and the orthologous genes of *B. oleracea* and *B. napus* are 42 and 59, respectively, but there are 94 *TH* genes in *B. napus*. In addition, by calculating the difference between the total *TH* genes in diploid species and the inherited *TH* genes, we found that many of the missing genes in *B. napus* belonged to the GT-2 subgroup, and most of the missing genes in *B. carinate* belonged to SIP1, but almost no genes were discarded in *B. juncea*. Compared with other subgroups, the genes in the GT-2 and SIP1 subgroups might produce more genes with new functions through duplication events after allotetraploidy. These genes with new functions may promote the growth and development of plants or improve the adaptability to their environment.

### 2.7. Interaction Analysis of BnaTH Genes

There are 21 *BnaTH* genes with interaction relationships, most of which belong to the GT-2 subgroup (14, 66.7%) (Figure S3). In the protein interaction network, we identified two hub genes, namely *BnaTH-94* and *BnaTH-41*, which can interact with eleven and eight *BnaTH* genes, respectively. In total, 28 miRNAs were found to target 35 *BnaTH* genes, and 73 pairs of interaction relationships were generated, among which the GT-2 (19, 54.3%) and SIP1 (12, 34.3%) subgroups accounted for the vast majority. Most miRNAs can target 1–3 *BnaTH* genes, but the miR396a can regulate 16 *BnaTH* genes (Figure S3). In addition, *BnaTH-19* can be regulated by nine miRNAs, which indicates that the function of this gene may be diverse.

### 2.8. Expression Patterns of BnaTH Genes in Different Tissues and under Abiotic Stresses

**Figure 8:**
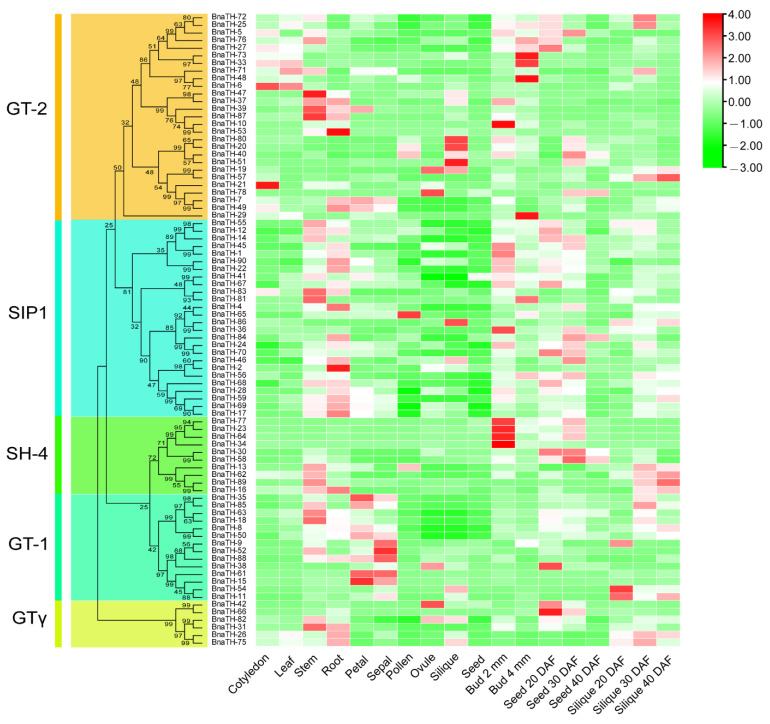
Expression pattern of *BnaTH* in different tissues and different developmental stages. The subgroup distribution and phylogenetic tree of BnaTH are shown on the left. FPKM values of *BnaTH* genes transformed by log2 were used in TBtools to construct heat maps. Expression levels are depicted by the different colors on the scale. Red and green represent high and low expression levels respectively.

In order to investigate the putative functions of *TH* genes in plant growth and development, we analyzed the expression patterns of *BnaTH* genes in nine different tissues (cotyledon, leaf stem, root, petal, sepal, pollen, ovule, silique, and seed) of *B. napus* and at different developmental stages of buds, seeds, and siliques (Figure 8). The results revealed that the expression profiles of the *TH* genes were diverse, but that, to a certain extent, the genes of the same subgroup displayed similar expression patterns. Overall, almost every tissue has gene expressions from five different subfamilies, but, in the same subfamily, the expression profiles are not exactly the same, indicating the different functions of the genes in the same subfamily. Seven genes (*BnaTH-76*, *BnaTH-27*, *BnaTH-33*, *BnaTH-25*, *BnaTH-73*, *BnaTH-29*, and *BnaTH-72*) belonging to the GT-2 subgroup are highly expressed in the cotyledons and leaves, which may mean that they play a role in the light responses of plants and in photosynthesis. With the growth of siliques, the expression of three genes (*BnaTH-89*, *BnaTH-19*, and *BnaTH-57*) in siliques also increased gradually, which may indicate involvement in the silique. We found that *BnaTH-49* was highly expressed in ovules and seeds, but weakly expressed in other tissues. Moreover, it was observed that the expression of *BnaTH-49* decreased with the maturation of seeds, which suggested that this gene might have negative regulation with seed maturation. In addition, a large number of *BnaTH* genes is abundantly expressed in stems, roots, buds, and most other tissues, which indicates that *TH* genes are generally involved in plant growth and development.

**Figure 9:**
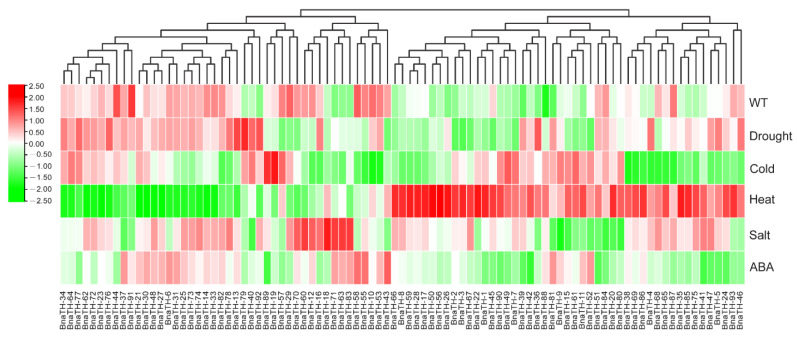
Expression patterns of *BnaTH* under different stresses. FPKM values of *BnaTH* genes transformed by log2 were used in TBtools to construct heat maps. Expression levels are depicted by the different colors on the scale. Red and green represent high and low expression levels respectively.

To determine the response of *BnaTH* genes to abiotic stresses, the expression levels of *BnaTH* genes in *B. napus* under drought stress, cold stress, heat stress, salt stress, and ABA treatment were analyzed (Figure 9). Expression profile analysis revealed that many *BnaTH* genes could be induced under different stresses. Most *TH* genes were significantly up- or down-regulated under heat treatment, implying that *TH* genes are generally responsive to heat stress and that they have different regulatory networks. Some genes showed similar expression patterns under different stress treatments. For example, the expression levels of *BnaTH-85*, *BnaTH-26*, *BnaTH-78*, *BnaTH-92*, and *BnaTH-4* were significantly increased after drought treatment and salt stress, indicating that these genes may be associated with multiple stress response processes. In addition, we found that some homologous genes showed similar expression patterns, such as *BnaTH-2* and *BnaTH-56*, while others had opposite expression profiles, such as *BnaTH-5* and *BnaTH-27*. Overall, most of the genes that can respond to abiotic stresses belong to GT-2 and SIP1 clades, which suggests that the GT-2 and SIP1 clade genes may be significantly expanded to adapt to the environment.

**Figure 10:**
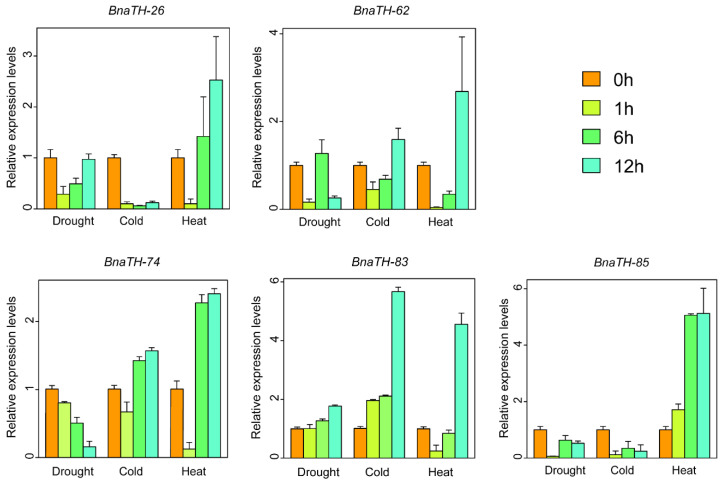
Expression profiles of five random selected *TH* genes in response to drought, cold, and heat treatments. Leaves were collected at 0 h, 1 h, 6 h, and 12 h after different treatments. Data were normalized to the *BnACTIN* gene. Vertical bars indicate standard deviations.

To validate the expression patterns of *TH* genes under drought, cold, and heat stresses, qRT-PCRs were used to detect the expression levels of five randomly selected *TH* genes (Figure 10). The differential expression profiles of these selected genes under different stresses imply that they have different functions. For example, under cold treatment, the expression of *BnaTH-26* decreased significantly at 1 h, and remained at low levels at 6 and 12 h, while the *BnaTH-83* continued to increase. The expression of *BnaTH-74* decreased gradually with the prolonged drought treatment, while *BnaTH-83* showed the opposite expression pattern. All five of the selected genes responded to heat stress. Moreover, interestingly, except for *BnaTH-85*, the expression levels of the genes decreased first and then increased after heat treatment. Thus, all five genes were induced by various abiotic stresses with differential expression levels.

## 3. Discussion

As sessile organisms, plants must endure various abiotic stresses, including drought, cold, high temperatures, salt, and other environmental factors. Transcription factor TH has been found to be involved in regulating plant growth and development, as well as responses to biotic and abiotic stresses. With the release of the genome sequences of many plants, genome-wide identification of *TH* genes has been carried out in many species. However, other than for around 10 reported *TH* genes (Table S10), the identification and the function of Brasscia *TH* genes, especially in *B. napus*, have not been well reported [5,16,17,18,27,28,29,30,31,32,33]. In this study, a total of 455 *TH* genes was identified in six Brassica species and *Arabidopsis*, and the phylogenetic relationship, structure, *cis*-acting elements, chromosome distribution, gene duplication events, and collinearity were investigated. Furthermore, the expression patterns of these *TH* genes in *B. napus* were analyzed using RNA-seq with the highly sensitive method of qRT-PCRs [34,35]. Through comparative genomics, we explored the evolution of the *TH* gene family in Brassica, and present comprehensive information about this gene family, which provides clues for the functional study of *TH* genes in *B. napus*.

### 3.1. The Retention and Loss of TH Genes in Brassica during Evolution

Previous studies have proposed that the genome of Brassica originated from a genome with a similar structure to *Arabidopsis* through three rearrangement variants and inherited from a common hexaploid ancestor [25,36]. After whole-genome duplication or triplication (WGD/WGT), plants generally tend to restore the number of genes to the diploid level through gene loss [37]. However, in Brassica, this is more suitable for collinear genes in conserved syntenic regions. It is estimated that 60% of the genome is lost after triplication, and the number of genes before triplication is almost recovered [26]. Massive gene loss and the frequent recombination of triplicated genomic blocks occurred in the Brassica genome after WGT, resulting in complex mosaics of triplicated ancestral genomic blocks in the A and C genomes [26]. *B. napus*, as a relatively young amphidiploid, has not undergone significant chromosome rearrangement since the genome fusion of the progenitors A and C. However, homologous recombination events between the two corresponding genomes are universal in newly shaped *B. napus* lines, and low levels of homologous recombination have been recognized in established canola cultivars [38,39,40]. Through comparative mapping of *B. napus* and *Arabidopsis*, three collinear fragments were usually identified in each diploid genome for every region of *Arabidopsis* studied [41,42].

In this study, 49 and 54 *TH* genes were identified in *B. rapa* and *B. oleracea*, respectively, while 94 *TH* genes were identified in *B. napus*. This situation also been observed in two other allotetraploids, *B. carinate* and *B. juncea*, indicating that some redundant *TH* genes were lost in the process of evolution. In order to explore the reasons for the loss of *TH* genes in tetraploid species, we took the number of *TH* genes in *Arabidopsis* as a reference, and first compared and analyzed the *TH* genes in diploid plants. Furthermore, we found that 20 *TH* genes in *Arabidopsis* have only one homologous gene in *B. rapa*, and five *TH* genes have two homologous genes in *B. rapa*. In *B. oleracea*, 19 *TH* genes correspond to a homologous gene with *Arabidopsis*, and 12 *TH* genes correspond evenly to six *Arabidopsis TH* genes. However, we found a large number of *AtTH* genes with more than one homologous gene in *B. nigra*; among these, 12 *AtTH* genes only found one homologous gene in *B. nigra*, but 11 *AtTH* genes found two homologous genes in *B. nigra*, and even *AtTH-24* corresponded to three homologous genes (*BniTH-9*, *-27*, *-46*) in *B. nigra*. This indicated that the genome of Brassica ancestors lost a large number of *TH* genes after WGT, and the number may tend to recover to its pre-tripling level. In addition, *B. nigra* retained more *TH* genes during the evolution of Brassica diploid species from hexaploid ancestors. In the collinearity analysis of *Arabidopsis* and Brassica tetraploid species, we found that most *TH* genes in *Arabidopsis* only found one or two homologous genes in each species. However, in every species of Brassica, a high proportion of *TH* segmental duplication genes was recognized, especially in tetraploid species, and most of these genes were from subgroups GT-2 and SIP1. To sum up, segmental duplication events may drive the expansion of *TH* genes in the evolution of Brassica species, and these duplicated genes may support plants to better adapt to various environments.

### 3.2. A Conserved Pattern of Motifs and TH Gene Structures

We confirmed that the conserved motifs and gene structures of 455 TH family members were consistent with the classifications of subfamilies. These subfamily members with similar gene structures and motif distributions may have arisen from gene duplication events and, therefore, they may have similar functions. Alternatively, these genes may have undergone pseudogenization, neofunctionalization, or subfunctionalization during evolution. We found that some motifs are present in all subgroups, while some motifs are almost unique to subfamilies, and these subgroup-specific motifs may perform a distinct function. In addition, our comparative analysis of the 10 conserved motifs of the TH proteins in *B. rapa*, *B. oleracea*, and *B. napus* revealed that almost all motifs in *B. napus* could find their counterparts in *B. rapa* or *B. oleracea*, but a small number of amino acid residues in these sequences were mutated, suggesting that *B. napus* inherited motifs from ancestral diploids in a relatively conserved state during hetero-tetraploidization.

### 3.3. Expression and Function Characteristics of TH Genes in B. napus

In the present study, we revealed the broad expression patterns of *TH* genes in *B. napus*; their transcripts expressed in almost all tissues and universally participated in the growth and development of *B. napus*, based on the RNA-seq data. Changes in *TH* gene expression patterns under abiotic stresses such as drought and osmotic stresses have been reported [16]. In our study, we determined the expression patterns of *TH* genes in *B. napus* seedlings exposed to five stresses to elucidate the roles of *TH* genes in response to adverse environmental conditions. The results showed that *TH* genes could be induced by various stresses, and the expression levels of some genes were significantly up-regulated after stresses; in contrast, *TH* genes could only be weakly induced by ABA. The genes *BnaTH-8*, *BnaTH-59*, and *BnaTH-28* were all abundantly expressed under heat stress, whereas the genes *BnaTH-4*, *BnaTH-80*, and *BnaTH-67* were differentially expressed under drought, cold, and salt stresses, respectively, indicating that these genes are involved in many biological processes in *B. napus* and respond to different stresses. The interaction relationships of BnaTH proteins and miRNA targeted *BnaTH* genes also revealed that *BnaTHs* regulated plant development and stress responses. In addition, the involvement of *cis*-acting elements in the expression of genes at different stages of plant growth and development and under different stresses has been widely reported. In our study, we identified a large number of stress-related *cis*-acting elements, such as MBS (drought), LTR (low temperature stress), and phytohormone response elements such as abscisic acid (ABRE), methyl jasmonate (CGTCA-motif and TGACG-motif), auxin (TGA element), salicylic acid (TCA element). Some phytohormones are important for plant stress resistance, such as ABA, which mediates the physiological response to stress induced by dehydration and salt stresses, suggesting that some hormone response elements not only affect plant growth and development but also have the potential to respond to stresses. Further qRT-PCR analysis verified that some genes were up-regulated under drought, cold, and heat stress, but most of them showed weak changes at the initial stage and significant differences at 12 h after the stress treatment (Figure 10).

## 4. Materials and Methods

### 4.1. Plant Materials

Seeds of *B. napus* Zhongshuang 9 (ZS9) were cultured in an illuminated incubator with a 16 h/8 h light dark cycle and a temperature of 22 °C. The seedlings were subjected to various stresses (drought, cold, and heat treatments) at the three-leaf stage. For the drought stress treatments, the seedlings were watered with a Hoagland nutrient solution containing 18% polyethylene glycol (PEG)-6000 (*w/v*). In the cold stress treatment, plants were kept in an illuminated incubator at 4 °C with the other conditions unchanged. Seedlings were also grown in an oven at 40 °C for heat stress treatment. We collected leaves from the plants treated with drought, cold, and heat stresses at 0 h, 1 h, 6 h, and 12 h after the beginning of treatment. After collection, all samples were frozen immediately in liquid nitrogen and stored at −80 °C for RNA extraction.

### 4.2. Identification and Characterization of the Trihelix (TH) Genes

We downloaded the entire genome sequence information of six Brassica species from the Brassica database (BRAD version 3.0; http://brassicadb.cn/, accessed on 18 July 2022) [43]. Additionally, the *Arabidopsis* genome information was acquired from Ensembl (http://plants.ensembl.org/, accessed on 18 July 2022) [44]. The Hidden Markov Model (HMM) profile (PF13837) downloaded from the Pfam database (http://pfam.xfam.org/, accessed on 18 July 2022) was utilized to search the trihelix domain (E-value < 1 × 10^−3^) by means of HMMER v3.0 software (http://hmmer.janelia.org/, accessed on 18 July 2022) [45,46]. We constructed seven species-specific HMM profiles, using the extracted protein domain sequences with E-value < 1 × 10^−28^ in the first search result. Then, we performed a second scan of the protein sequence (E-value < 1 × 10^−3^) to identify the *TH* genes based on the newly created species-specific HMM profiles. The output putative TH protein sequences were further authenticated via the online websites Pfam (http://pfam.xfam.org/, accessed on 19 July 2022) and SMART (https://smart.embl.de/, accessed on 19 July 2022) [47,48]. The basic physical and chemical characteristics of the candidate TH proteins, including molecular weight (MW), theoretical isoelectric point (pI), the aliphatic index (AI), the instability index (II), and the grand average of hydropathicity (GRAVY), were calculated using the ProtParam program (https://web.expasy.org/protparam/, accessed on 20 July 2022) [49]. In addition, the subcellular localization of TH proteins was predicted using WOLF PSORT (https://wolfpsort.hgc.jp/, accessed on 20 July 2022) [50].

### 4.3. Phylogenetic Analysis and Classification

Multiple sequence alignment of the AtTH, BcaTH, BjuTH, BnaTH, BniTH, BolTH, and BraTH protein sequences was performed using the ClustalX v2.1 program. Then, an unrooted phylogenetic tree was constructed via the neighbor-joining (NJ) method using MEGA 7.0 software with 1000 bootstrap repetitions [51]. The phylogenetic tree was visualized using Evolview software (https://evolgenius.info//evolview-v2/#login, accessed on 20 July 2022) [52].

### 4.4. Gene Structure and Conserved Motif Analysis

The exon-intron structure information was attained from the annotation file (gff3) of the sequenced genome. The online software MEME (https://meme-suite.org/, accessed on 21 July 2022) [53] was used to investigate the conserved motifs of TH proteins; the parameters were set with the maximum number of motifs as 10 and the motif width as 5–200 amino acids. TBtools was utilized to visualize the map of the exon–intron structures and motifs [54].

### 4.5. Analysis of Cis-Acting Elements in the Promoter Region

The PlantCARE (https://bioinformatics.psb.ugent.be/webtools/plantcare/html/, accessed on 22 July 2022) database was utilized to calculate the *cis*-acting elements within the 1500 bp sequence upstream of the transcription start site of the *TH* genes [55]. GSDS online software (http://gsds.gao-lab.org/, accessed on 22 July 2022) was employed to visualize the distribution map of *cis*-acting elements [56].

### 4.6. Chromosomal Distribution, Gene Duplication (Collinear), and Evolution of TH Genes

The chromosome location data for the *TH* genes were obtained from the genomic annotation file GFF3 of the seven species, then MapChart software [57] was used to locate the distribution of the *TH* genes on the chromosome, respectively. The analysis of the intra-species collinearity of *TH* genes in seven species was performed using collinear scanning toolkits (McScanX) [58], and the collinearity of gene pairs was presented using Circos v0.55 [59]. Moreover, McScanX software was used to analyze and plot collinearity between species.

### 4.7. Ka, Ks, and Ka/Ks Analysis

The synonymous (*Ks*) and nonsynonymous (*Ka*) substitution rates, and the selection pressure *Ka/Ks* ratio of homologous gene pairs and tandem array genes (adjacent *TH* genes on individual chromosomes), were calculated using TBtools [54].

### 4.8. Interaction Analysis of BnaTHs

The interaction of BraTH proteins was predicted using the STRING website (https://cn.string-db.org/, accessed on 15 September 2022). The potential miRNA-regulating sites of *BnaTH* genes were analyzed using psRNATarget [60]. The interaction relationships of BnaTH was visually edited using Cytoscape 3.8.2 [61].

### 4.9. RNA Isolation and qRT-PCR Analysis

Total RNA was extracted from leaves treated with different abiotic stresses using an RNA Prep Pure Plant Kit (Tiangen Biochemical Technology (Beijing, China) Co., Ltd.: DP201101X). The concentration and purity of the RNA were determined using a Nanodrop micro spectrophotometer (Thermo Scientific, Waltham, MA, USA) and agarose gel electrophoresis. The first strand of cDNA derived from mRNA was synthesized using HiScript^®^Q RT SuperMix (Vazyme, Nanjing, China). Quantitative real-time PCR (qRT-PCR) was carried out by SYBR-green fluorescence with a QuantStudio^TM^ Real-Time PCR System (Thermo Fisher Scientific). Five *TH* genes were randomly selected to validate the expression patterns under drought, cold, and heat stresses (Table S11). The data were normalized by the internal control gene *BnACTIN* (*BnaA03g55890D*) and the 2^−ΔΔCT^ analysis method was utilized to calculate relative expression levels [62].

### 4.10. Expression Profile Analysis

To explore the spatiotemporal expression patterns of rapeseed under different tissues and stresses, the relative FPKM value (fragments per kilobase of transcript per million fragments mapped) of RNA-seq data, PRJNA389508 (seedlings treated with five stress conditions) and PRJNA311067 (seeds harvested at different times after pollination), were retrieved in the BrassicaEDB website (https://brassica.biodb.org/downloads, accessed on 20 August 2022), and the expression levels of different tissues (cotyledon, leaf, stem, root, petal, sepal, etc.) were acquired from the *Brassica napus* information resource (http://yanglab.hzau.edu.cn/BnIR/expression_zs11, accessed on 20 August 2022) [63,64,65,66]. The FPKM values were log2 transformed and the heat map of hierarchical clustering was visualized using TBtools software [54].

## 5. Conclusions

In summary, a total of 455 *TH* genes was identified in six Brassica species and *Arabidopsis*. The evolutionary history of the *TH* genes was explored by integrating phylogenetic analysis, conserved motif identification, gene duplication event analysis, collinearity analysis between six Brassica species and *Arabidopsis*, and comparative genomic analysis. Many of the missing genes in *B. napus* belonged to the GT-2 subgroup, and most of the missing genes in *B. carinate* belonged to SIP1, but almost no genes were discarded in *B. juncea*. In addition, some mutations occurred in the motifs of TH proteins following hetero-tetraploidization in *B. napus*, with changes in the sequences of their amino acid residues. Expression pattern analysis revealed that *BnaTH* genes have different expression patterns, which can be induced by various stresses. Further transgenic validation or CRISPR/Cas9 knock out of these *BnaTH* genes could reveal their roles in stresses. In this study, a systematic analysis of *TH* genes in Brassica species, including their origin and evolution, and the function of *BnaTH* genes, was carried out to provide insights for further studies on the function of *TH* genes in *B. napus*.

## Data Availability

The transcriptome data are available from the online website BrassicaEDB (https://brassica.biodb.org/downloads accessed on 20 August 2022, PRJNA389508 and PRJNA311067) and Brassica napus information resource (http://yanglab.hzau.edu.cn/BnIR/expression_zs11, accessed on 20 August 2022).

## Supplementary Materials

The following supporting information can be downloaded at: https://www.mdpi.com/article/10.3390/ijms232415766/s1.
