# Supplementary figures and images for: Conservation and Divergence of the Trihelix Genes in Brassica and Expression Profiles of BnaTH Genes in Brassica napus under Abiotic Stresses
Source: Int J Mol Sci. 2022 Dec 12;23(24):15766. doi: 10.3390/ijms232415766 (PMC9779230; doi:10.3390/ijms232415766)

SH4  
GTy  
GT-1  
GT-2  
SIP1

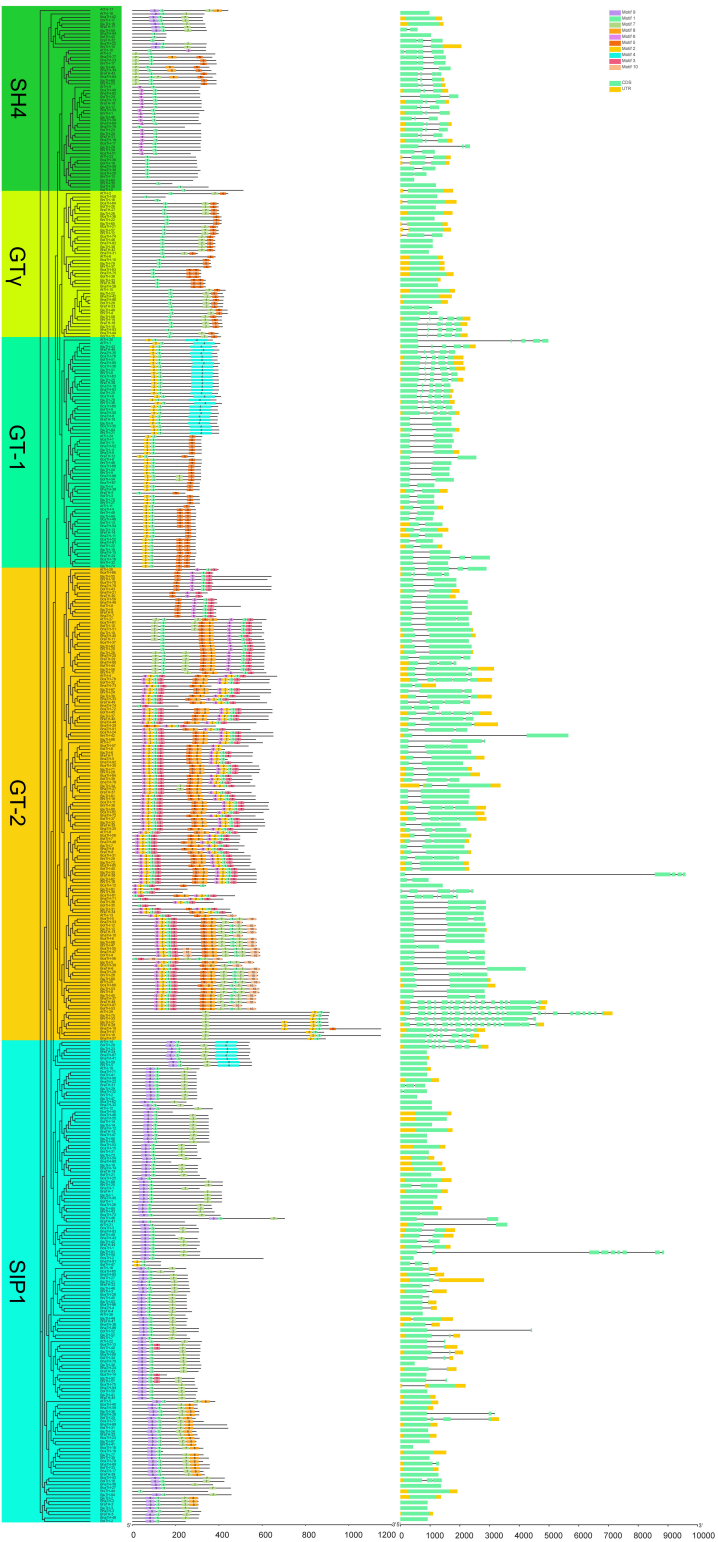

Supplement: Supplementary file 1 [file ijms-23-15766-s001.zip › Figure S1.pdf]

## A 16 Bra SIP1 TH

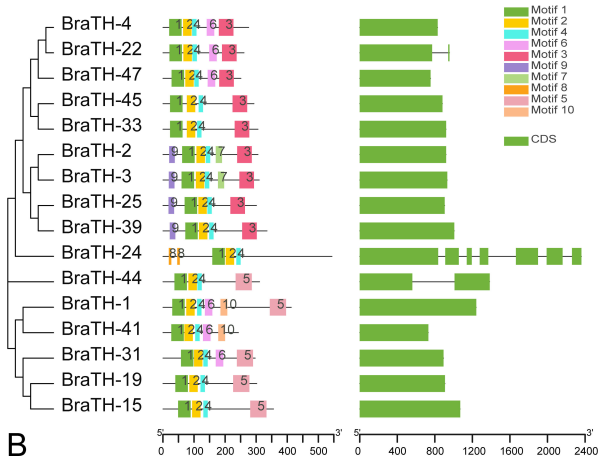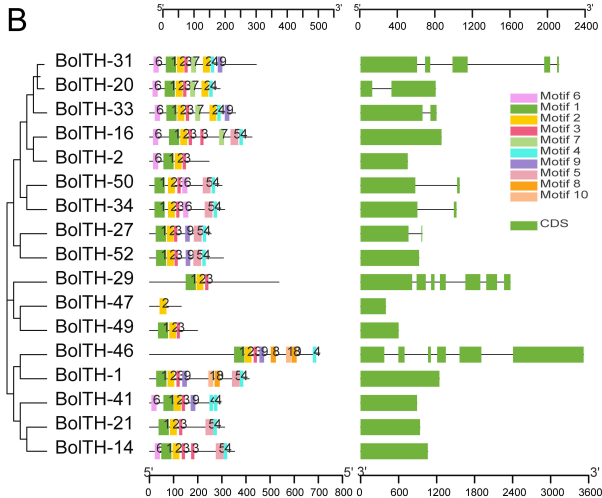

## C 30 Bna SIP1 TH

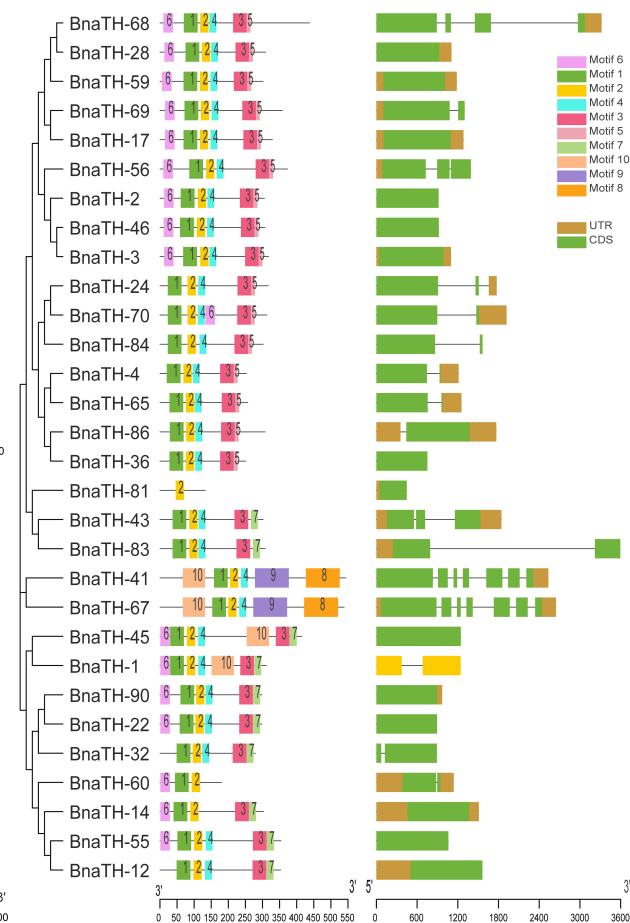

## 17 BoI SIP1 TH

Supplement: Supplementary file 1 [file ijms-23-15766-s001.zip › Figure S2.pdf]

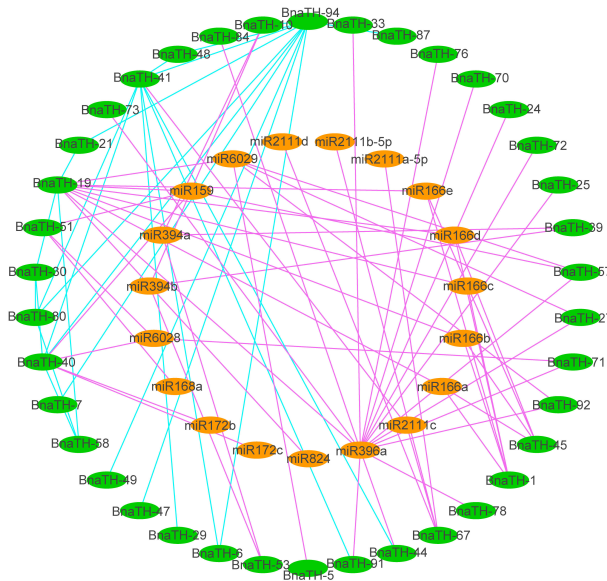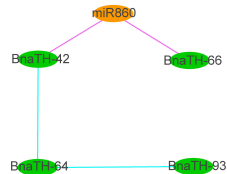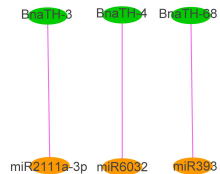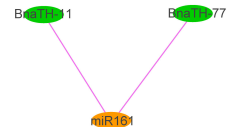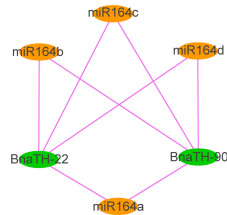

Supplement: Supplementary file 1 [file ijms-23-15766-s001.zip › Figure S3.pdf]
